# The Human Adenovirus Type 5 E4orf6/E1B55K E3 Ubiquitin Ligase Complex Can Mimic E1A Effects on E2F

**DOI:** 10.1128/mSphere.00014-15

**Published:** 2015-11-11

**Authors:** Frédéric Dallaire, Sabrina Schreiner, G. Eric Blair, Thomas Dobner, Philip E. Branton, Paola Blanchette

**Affiliations:** a Department of Biochemistry, McGill University, Montreal, Québec, Canada; b Institute of Virology, Technische Universität München/Helmholtz Zentrum München, Munich, Germany; c School of Molecular and Cellular Biology, University of Leeds, Leeds, United Kingdom; d Heinrich Pette Institute, Leibniz Institute for Experimental Virology, Hamburg, Germany; e Department of Oncology, McGill University, Montreal, Québec, Canada; f Goodman Cancer Research Centre, McGill University, Montreal, Québec, Canada; University of Michigan

**Keywords:** adenovirus, ubiquitin ligase, E4orf6, E1B55K, E1A

## Abstract

The human adenovirus E4orf6/E1B55K E3 ubiquitin ligase is well known to promote viral replication by degrading an increasing number of cellular proteins that inhibit the efficient production of viral progeny. We report here a new function of the adenovirus 5 (Ad5) viral ligase complex that, although at lower levels, mimics effects of E1A products on E2F transcription factors. When expressed in the absence of E1A, the E4orf6 protein in complex with E1B55K binds E2F, disrupts E2F/retinoblastoma protein (Rb) complexes, and induces hyperphosphorylation of Rb, leading to induction of viral and cellular DNA synthesis as well as stimulation of early and late viral gene expression and production of viral progeny of E1/E3-defective adenovirus vectors. These new and previously undescribed functions of the E4orf6/E1B55K E3 ubiquitin ligase could play an important role in promoting the replication of wild-type viruses.

**IMPORTANCE** During the course of work on the adenovirus E3 ubiquitin ligase formed by the viral E4orf6 and E1B55K proteins, we found, very surprisingly, that expression of these species was sufficient to permit low levels of replication of an adenovirus vector lacking E1A, the central regulator of infection. E1A products uncouple E2F transcription factors from Rb repression complexes, thus stimulating viral gene expression and cell and viral DNA synthesis. We found that the E4orf6/E1B55K ligase mimics these functions. This finding is of significance because it represents an entirely new function for the ligase in regulating adenovirus replication.

## Abstract (precis)

During the course of work on the adenovirus E3 ubiquitin ligase formed by the viral E4orf6 and E1B55K proteins, we found, very surprisingly, that expression of these species was sufficient to permit low levels of replication of an adenovirus vector lacking E1A, the central regulator of infection. E1A products uncouple E2F transcription factors from Rb repression complexes, thus stimulating viral gene expression and cell and viral DNA synthesis. We found that the E4orf6/E1B55K ligase mimics these functions. This finding is of significance because it represents an entirely new function for the ligase in regulating adenovirus replication.

## INTRODUCTION

E4orf6 and E1B55K proteins of all human adenoviruses (Ads) form functional cullin-based E3 ubiquitin ligase complexes that enhance viral replication by degrading a growing number of cellular proteins (1–3). The E4orf6 protein associates via multiple BC boxes with cellular elongins B and C to facilitate binding of either Cul5 or Cul2 and other components to form the core ligase complex (2, 4–7). E1B55K associates with E4orf6 only when present in the ligase complex, as E4orf6 BC box mutants do not interact with E1B55K (6). E1B55K is believed to function as the major substrate acquisition component of the ligase through interactions with a number of degradation targets, including p53 (7, 8), Mre11 (6, 9), DNA ligase IV (10), integrin α3 (11), Bloom helicase (12), ATRX (13), Tip60 (14), and SPOC1 (13). E1B55K alone has been shown to degrade Daxx by ubiquitin-mediated degradation (15), whereas E4orf6 of adenovirus 12 (Ad12) (but no other serotype) degrades TOPBP1 via an unknown mechanism (16–18). Considerable variation in the pattern of protein degradation exists among human adenovirus serotypes: thus far, DNA ligase IV is the only substrate found to be targeted by all (2). Ad5 viral mutants defective in either E4orf6 or E1B55K replicate poorly (19, 20), and their complex has been implicated in a number of important functions, including regulation of the stability of p53 (7, 8, 21–24) and the selective transport of late viral mRNAs (19, 25–28); however, additional functions could exist that enhance viral replication.

It has been known for decades that the major regulators of human adenovirus replication are the products of early region 1A (E1A), which encodes a series of species, notably products of the 13S (289R) and 12S (243R) E1 mRNAs (29). Both interact with the retinoblastoma (Rb) protein and other members of the “pocket” protein family that regulate E2F transcription factors through complex formation with transcriptional repression complexes (30, 31). Binding by E1A products frees E2F to induce expression of both early viral transcripts and cell cycle genes that regulate the initiation of DNA synthesis, thus permitting replication even in terminally differentiated epithelial cells, the major targets of human adenoviruses (32–34). In addition, E1A 289R contains a transcriptional activation domain that promotes transcription of early viral genes (35).

In the present studies, we noted that when Ad5 E4orf6 and E1B55K were expressed from an adenovirus vector lacking the E1 region (E1A and E1B), viral DNA replication was induced as well as expression of early and late viral gene products to yield progeny virions. We found that the E4orf6/E1B55K complex activates E2F by inducing hyperphosphorylation of Rb and disrupting the Rb/E2F complex, indicating a new function for the ligase complex. Although these effects were far less efficient than those induced by E1A products, it is possible that the viral ligase may contribute to the E1A-dependent activation of E2F in wild-type infections to enhance viral replication.

## RESULTS

### E4orf6 and E1B55K cooperate to induce viral replication in the absence of E1A.

**FIG 1:**
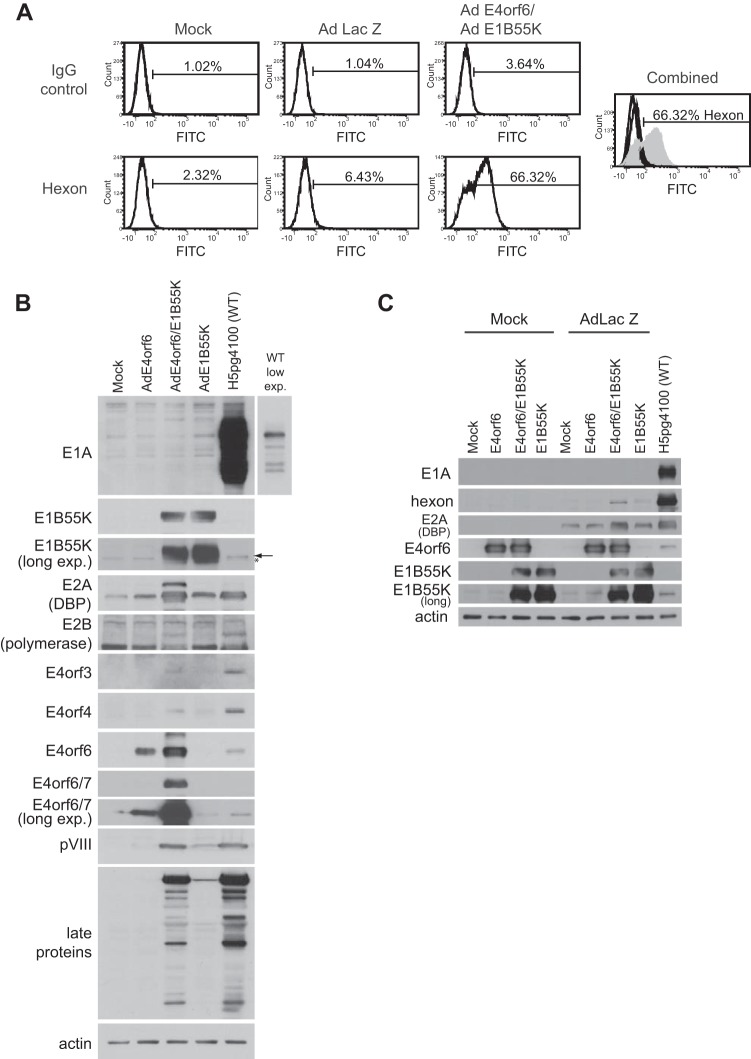
Infection with AdE4orf6 and AdE1B55K induces expression of viral proteins. (A) H1299 cells were infected for 48 h with the indicated viral vectors at an MOI of 35 and complemented with AdLacZ as required for a total MOI of 70. Cells were lifted, fixed, and stained intracellularly with antibody against hexon before being examined by flow cytometry. The plot on the right labeled “combined” consists of all of the previous plots combined in one, with the hexon plot for AdE4orf6/E1B55K shaded in gray. Long exp., long exposure. (B) Cells were infected as in panel A or with wild-type virus at an MOI of 5, and whole-cell extracts were immunoblotted for several viral proteins, as indicated, using the appropriate antibodies. The arrow in panel B indicates the position of migration of E1B55K. (C) H1299 cells were either mock infected or infected with AdLacZ at an MOI of 70 or wild-type virus at an MOI of 5 and transfected with the indicated plasmid DNAs for 48 h. Whole-cell extracts were immunoblotted as indicated using the appropriate antibodies. An asterisk denotes a background band.

In our studies on the Ad5 E4orf6/E1B55K E3 ubiquitin ligase complex, we have sometimes employed nonreplicating Ad5-based vectors that lack the E1 region (E1A and E1B) but express either E4orf6 or E1B55K at high levels from a cytomegalovirus (CMV) promoter, although very small amounts of E2, E3, and E4 proteins are produced from the vector genome (our unpublished results and see reference 36). In recent experiments, we noted some cytopathic effect when H1299 human small cell lung carcinoma cells were coinfected with Ad vectors expressing E4orf6 (AdE4orf6) and E1B55K (AdE1B55K), but not with either vector alone or with the control vector expressing LacZ (AdLacZ). As these observations suggested that replication of the viral vectors might have occurred, we analyzed vector-infected cells by immunostaining followed by flow cytometry using an antibody against the Ad5 late hexon protein to determine if in fact late viral proteins were being formed. Figure 1A shows that, whereas very few cells expressed hexon in AdLacZ- and mock-infected cells, a significant number of cells expressed hexon in cells coinfected with AdE4orf6 and AdE1B55K. Our initial concern was that a vector stock might be contaminated with low levels of wild-type Ad5, and thus we undertook by Western blotting using the appropriate antibodies a thorough examination of the expression of both early and late Ad5 proteins under these conditions. Figure 1B shows, using wild-type (WT) Ad5 (H5*pg*4100)- and mock-infected cells as a reference, that as expected E4orf6 and E1B55K proteins were expressed from AdE4orf6 and AdE1B55K, respectively. In no case other than in wild-type-infected cells were detectable levels of E1A products present, indicating the absence of contaminating wild-type virus in the vector stocks; however, significant levels of E2 products E2A-72K DNA binding protein (DBP) and E2B DNA polymerase and E4 products E4orf3 and E4orf4, as well as late products pVIII and capsid proteins, were present in AdE4orf6/AdE1B55K-infected cells. In some cases, very low levels of some of these products were evident in AdE1B55K-infected cells; however, as mentioned previously, this effect was probably due to low levels of E4orf6 expression from the vector genome (36). In these viral vectors, the majority of the E3 region has been deleted (37), and thus probing for the E3 products showed that, as expected, only the gp19K protein was expressed (data not shown). These findings also confirmed the absence of any contaminating wild-type virus in our preparations. To demonstrate that these effects were not dependent solely on expression of E4orf6 and E1B55K from viral vectors, either mock- or AdLacZ-infected cells were transfected with plasmid DNAs expressing E4orf6 and/or E1B55K and analyzed for hexon protein and DBP production. Figure 1C shows that when E4orf6 and E1B55K were coexpressed in AdLacZ-infected cells, hexon protein was again produced, although in smaller amounts than in Fig. 1B. This decrease could possibly be due to the fact that lower levels of expression of E4orf6 and E1B55K took place, due in part to the fact that a lower percentage of cells express these proteins than in vector-infected cultures. Nevertheless these results clearly indicated that in combination, E4orf6 and E1B55K are capable of inducing significant levels of early and late viral proteins.

**FIG 2:**
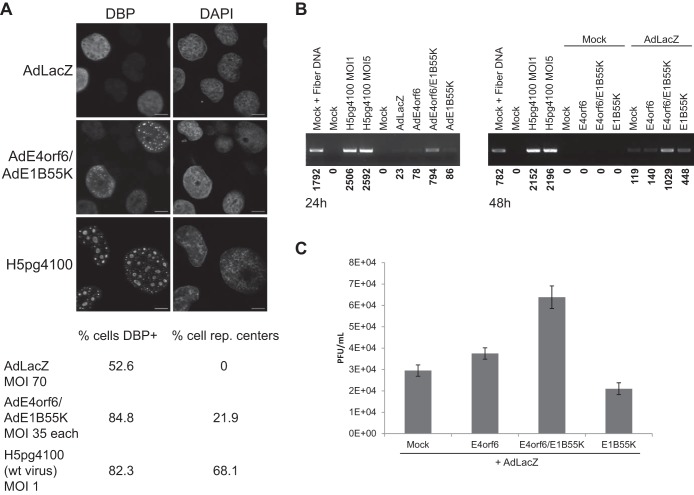
Infection with AdE4orf6 and AdE1B55K induces viral replication. (A) H1299 cells were infected with AdLacZ at an MOI of 70, AdE4orf6 and AdE1B55K at an MOI of 35 each, or with wild-type virus at an MOI of 1 for 24 h, fixed in methanol, and stained for DBP and DNA (DAPI). rep. centers, replication centers. (B) H1299 cells were infected with the viral vectors indicated at an MOI of 35 and complemented with AdLacZ as required for a total MOI of 70 or with wild-type virus at an MOI of 1 or 5 for 24 h (left panel). In the right panel, cells were either mock infected or infected with AdLacZ at an MOI of 70 or wild-type virus at an MOI of 5 and transfected with the indicated plasmid DNAs for 48 h. PCR for the fiber gene was performed on the lysates. Numbers below the panel indicate quantification results by ImageJ of the PCR bands. (C) Cells were infected as in panel B for 48 h and lysed, and the titer was determined by plaque assay on 293 cells.

**FIG 3:**
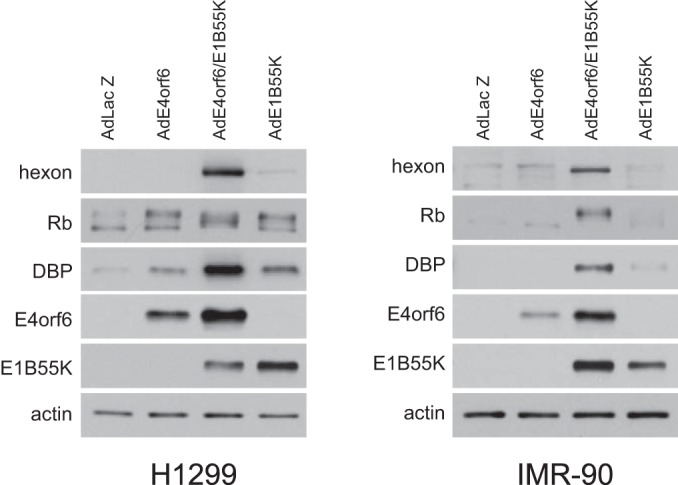
AdE4orf6 and AdE1B55K effects in normal IMR-90 cells. H1299 and IMR-90 cells were infected for 48 h with the indicated viral vectors at an MOI of 35 and complemented with AdLacZ as required for a total MOI of 70. Whole-cell extracts were immunoblotted for a series of proteins as indicated using the appropriate antibodies.

To demonstrate that a full productive infection was achieved, several properties of the infectious cycle were examined. Adenovirus infection involves formation of viral replication centers in which viral DNA synthesis occurs (38). Thus, at 24 h postinfection (p.i.), AdLacZ (multiplicity of infection [MOI], 70 PFU/cell)-, AdE4orf6/AdE1B55K (MOI, 35 PFU/cell)-, and Ad5 H5*pg*4100 wild-type (MOI, 1 fluorescent focus-forming unit [FFU])-infected cells were analyzed by confocal fluorescence microscopy following immunostaining with anti-DBP antibodies and treatment with 4′,6-diamidino-2-phenylindole (DAPI). Figure 2A shows that whereas a low level of DBP was expressed in some cells (52.6%), in AdLacZ-infected cells, over 80% of both AdE4orf6/AdE1B55K- and wild-type-infected cultures expressed detectable levels of DBP, and both contained structures typical of replication centers, although the percentage of cells was higher with wild-type virus. To determine if replication of viral DNA took place, cells infected with wild-type virus or viral vectors were harvested at 24 h p.i. and extracts were analyzed using a semiquantitative PCR-based assay in which the levels of DNA encoding fiber protein were measured. Figure 2B (left panel) shows that in addition to wild-type-infected cells, only those infected with both AdE4orf6 and AdE1B55K showed an increase in DNA level. Figure 2B (right panel) shows a similar result in which E4orf6 and E1B55K expressed from transfected plasmid DNAs in AdLacZ-infected cells induced viral DNA replication. Figure 2C shows that induction of viral DNA and protein synthesis by E4orf6/E1B55K also led to production of infectious progeny virions, as measured by plaque assays of extracts from cells infected by the control AdLacZ viral vector and transfected with plasmid DNAs expressing these proteins. Thus, E4orf6 and E1B55K appeared capable of inducing a full virus infectious cycle in the absence of E1A, although clearly not as efficiently as in wild-type-infected cultures. As these studies were performed in H1299 tumor cells, the ability of the E4orf6 and E1B55K vectors to induce viral replication was also tested in more normal noncancerous human cells to better reflect the conditions typical of adenovirus infections. As shown in Fig. 3A, IMR-90 cells (lung fibroblasts, right panel) were infected in parallel to H1299 cells (left panel). Late hexon and DBP proteins were produced following coinfection by the viral vectors in both IMR-90 and H1299 cells. These findings suggested that the effects observed following expression of the ligase proteins were not the result of physiological conditions unique to cancer cells.

### Induction of viral replication requires formation of the E4orf6/E1B55K ligase complex.

**FIG 4:**
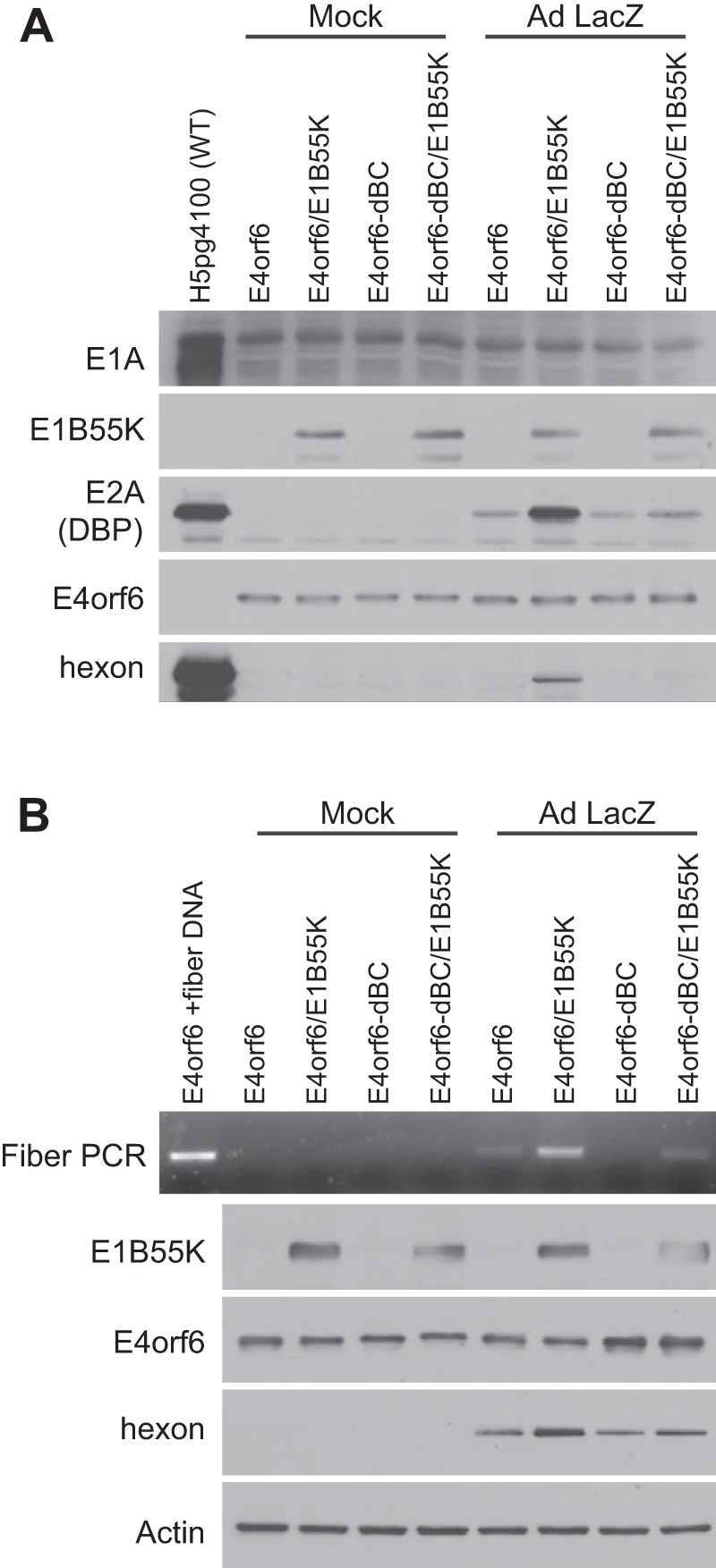
Effect of E4orf6/E1B55K is dependent on ligase complex formation. (A) H1299 cells were infected/transfected as in Fig. 1D for 48 h, and whole-cell extracts were immunoblotted as indicated using the appropriate antibodies. (B) H1299 cells were infected/transfected and processed as in panel A, but PCR for the fiber gene was also performed on the lysates.

To determine if induction of viral replication requires formation of the Ad5 E4orf6/E1B55K Cul5-based ligase complex, studies were carried out employing cDNAs expressing a mutant form of E4orf6 (E4orf6-dBC) in which the BC box sequence had been altered such that it could no longer bind to elongins B and C and thus was incapable of forming the ligase complex (6). Studies similar to those in Fig. 1D were carried out in mock- or AdLacZ-infected H1299 cells transfected with combinations of plasmid DNAs expressing E4orf6, E4orf6 and E1B55K, E4orf6-dBC, or E4orf6-dBC and E1B55K, and cell extracts were analyzed by Western blotting using antibodies against DBP or capsid proteins. Figure 4A shows that DBP and late proteins (notably hexon) were produced only in AdLacZ-infected cells expressing both wild-type E4orf6 and E1B55K and not with the E4orf6-dBC mutant. (Note that the small amounts of DBP in controls are probably due to low levels of DBP expressed from the LacZ viral vector.) To examine this question further, induction of viral DNA synthesis was measured in similar cultures using the semiquantitative PCR-based fiber DNA assay. Figure 4B shows that only in the case of expression of wild-type E4orf6 and E1B55K did DNA levels increase above the small amounts present from the infecting AdLacZ vector DNA with a corresponding increase in hexon protein. Thus, induction of viral replication required formation of the E4orf6/E1B55K ligase complex.

### The E4orf6/E1B55K complex activates viral E2 promoter.

**FIG 5:**
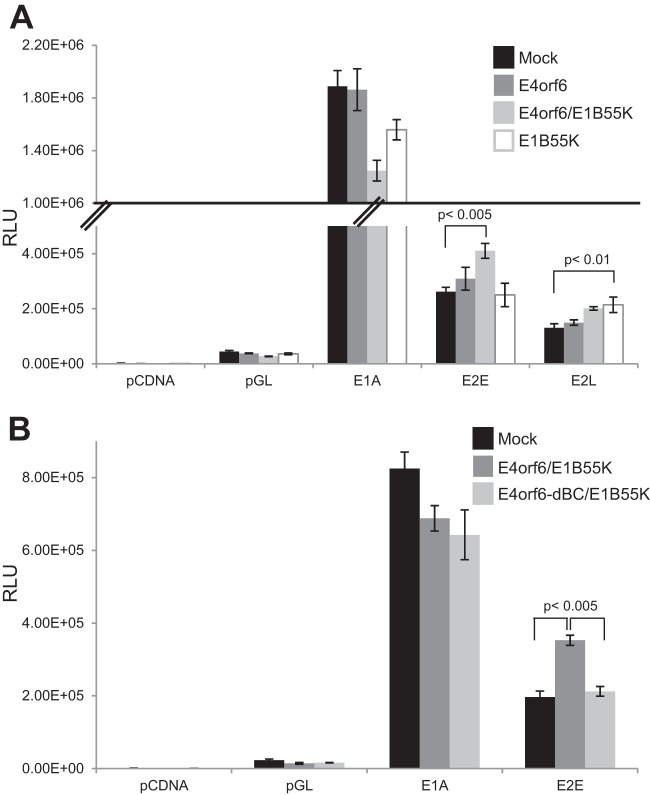
E4orf6/E1B55K activates the viral E2 promoter. (A and B) H1299 cells were transfected with plasmid DNAs expressing the indicated luciferase reporter constructs, the *Renilla* luciferase control, and E4orf6 or E1B55K for 24 h. Lysates were used for luciferase assays. RLU, relative luminescence units.

To begin to analyze the mechanism of induction of viral replication, studies were carried out to determine if the E4orf6/E1B55K complex was capable of inducing expression from viral promoters. Cells were transfected with plasmid DNAs expressing E4orf6 and E1B55K along with those expressing luciferase reporter constructs regulated by each viral promoter (13) or control pGL promoters. Figure 5A shows results obtained with the E1A, E2E, and E2L viral promoters, with those of E1A being representative of our findings with all other viral promoters (data not shown). In the case of the E1A promoter, expression of the reporter gene was somewhat reduced relative to that in controls following expression of E4orf6 and E1B55K or E1B55K alone. With the E2E reporter, E4orf6 or E1B55K alone had little effect; however, when expressed together, they produced a statistically significant increase in transcription (*P* < 0.005). With the E2L reporter construct, E4orf6 alone had no effect; however, E1B55K alone induced a consistent significant increase in expression (*P* < 0.01) that was not increased by coexpression of E4orf6. Figure 5B shows that the increase in E2E expression did not occur when E1B55K was coexpressed with the E4orf6-dBC mutant, indicating a requirement to form the E4orf6/E1B55K ligase complex.

### The E4orf6/E1B55K complex activates E2F-dependent transcription.

**FIG 6:**
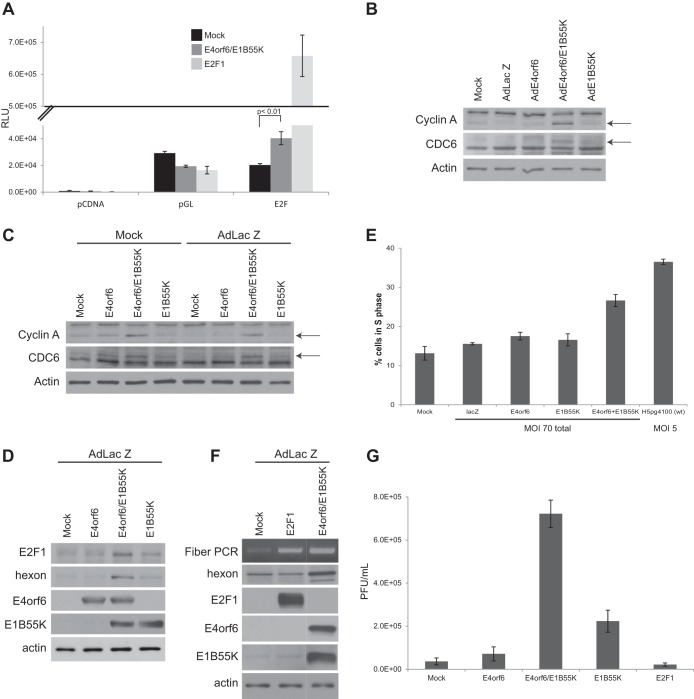
Effect of E4orf6/E1B55K on viral replication is mediated in part through activation of E2F. (A) H1299 cells were transfected with control or E2F-responsive luciferase reporter construct, the *Renilla* luciferase control, and cDNAs expressing E4orf6, E1B55K, or E2F1 for 24 h, and lysates were used for luciferase assays. RLU, relative luminescence units. (B) H1299 cells were infected with the viral vectors indicated at an MOI of 35 and complemented with AdLacZ as required for a total MOI of 70, and whole-cell extracts were immunoblotted as indicated using the appropriate antibodies. (C) H1299 cells were either mock infected or infected with AdLacZ at an MOI of 70 and transfected with the indicated plasmid DNAs for 48 h. Whole-cell extracts were immunoblotted as indicated using the appropriate antibodies. (D) H1299 cells were infected with AdLacZ at an MOI of 70 and transfected with the indicated plasmid DNAs for 48 h. Whole-cell extracts were immunoblotted as indicated using the appropriate antibodies. (E) H1299 cells were infected as in Fig. 1B for 48 h, fixed, PI stained, and analyzed by flow cytometry. (F) H1299 cells were infected with AdLacZ at an MOI of 70 and transfected with the indicated plasmid DNAs for 48 h. Whole-cell extracts were immunoblotted as indicated using the appropriate antibodies, and PCR for the fiber gene was performed. (G) H1299 cells were infected/transfected as in panel E for 48 h, and the titer was determined by plaque assay on 293 cells. Arrows in panels B and C indicate the positions of gel migration for relevant proteins.

Previous studies showed that expression of the E2E promoter is highly dependent on transcription factor E2F1 (39, 40). To determine directly if the E4orf6/E1B55K complex enhances expression of E2F-dependent promoters, studies were conducted using H1299 cells cotransfected with a plasmid DNA encoding a luciferase reporter construct containing four E2F1 binding sites, pGL-E2F (41). Figure 6A shows that overexpression of E2F1 induced a major increase in expression; however, coexpression of E4orf6 and E1B55K also induced a significant increase relative to controls. We also analyzed cell extracts by Western blotting for the endogenous levels of two proteins known to be encoded by E2F-dependent genes, cyclin A and CDC6 (42, 43). Figure 6B shows that only cells coinfected with AdE4orf6 and AdE1B55K exhibited increased levels of these species. Figure 6C shows a similar effect when E4orf6 and E1B55K were expressed following transfection of plasmid DNAs in both mock- and AdLacZ-infected cells. We also examined expression of endogenous E2F1 protein following transfection of plasmid DNAs in AdLacZ-infected cells. As seen in Fig. 6D, E2F1 levels also increased following expression of E4orf6 and E1B55K. As activation of E2F is known to promote the G_1_/S cell cycle transition, H1299 cells were infected with wild-type Ad5 or adenovirus vectors, and at 48 h p.i., cells were stained with propidium iodide (PI) and analyzed by flow cytometry to determine the percentage of cells in S phase. Figure 6E shows that although wild-type adenovirus infection caused the greatest increase in S-phase cells, coinfection with AdE4orf6 and AdE1B55K also resulted in a major increase. These results indicated that the E4orf6/E1B55K complex induces both viral and cellular DNA synthesis. It was of interest to compare levels of E4orf6/E1B55K-induced viral DNA synthesis and production of late viral proteins and progeny virions relative to those obtained through overexpression of E2F1. Thus, AdLacZ-infected cells were transfected with plasmid DNAs encoding E2F1 or E4orf6 and E1B55K, and cell extracts were analyzed for viral DNA synthesis using the semiquantitative fiber DNA PCR-based assay and for late proteins by Western blotting. Figure 6F shows that overexpression of both E2F1 and E4orf6/E1B55K induced significant viral DNA replication; however, only expression of E4orf6/E1B55K resulted in synthesis of late viral proteins. These results indicated that the E4orf6/E1B55K complex not only acts to induce viral DNA synthesis but also contributes additional functions to produce late viral products. This effect was also evident in virus formation as Fig. 6G shows that significant plaque-forming progeny were produced only by expression of E4orf6/E1B55K and not by overexpression of E2F1. This effect is not surprising as the E4orf6/E1B55K complex is known to promote viral replication in several ways, including inducing the selective transport of late viral mRNAs (19, 25–28).

### The E4orf6/E1B55K complex promotes Rb hyperphosphorylation.

**FIG 7:**
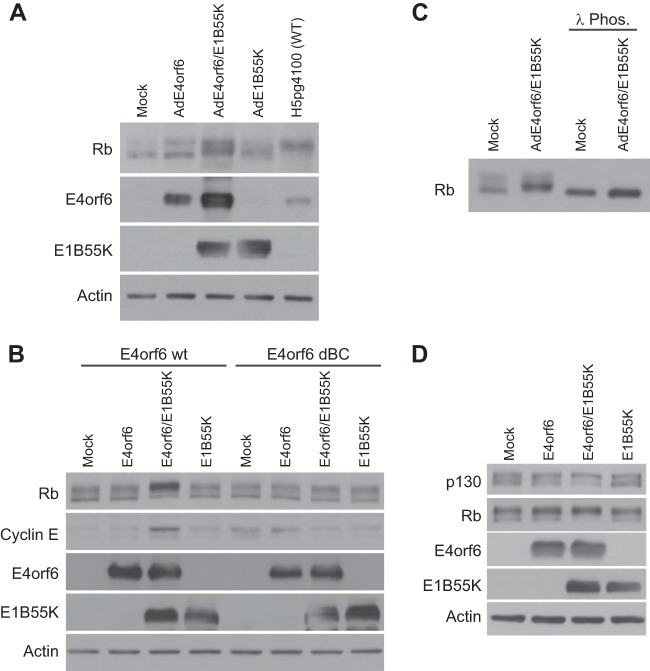
Phosphorylation state of pocket proteins is affected by E4orf6/E1B55K. (A) H1299 cells were infected with viral vectors indicated at an MOI of 35 and complemented with AdLacZ as required for a total MOI of 70 or with wild-type virus at an MOI of 5 for 48 h. Whole-cell extracts were immunoblotted as indicated using the appropriate antibodies. (B) H1299 cells were infected with AdLacZ at an MOI of 70 and transfected with the indicated plasmid DNAs for 48 h. Whole-cell extracts were immunoblotted as indicated using the appropriate antibodies. (C) H1299 cells were infected with indicated vectors as in panel A. Whole-cell extracts present in the two lanes at the right were treated with lambda phosphatase (Phos.) prior to separation on gels, and all were immunoblotted with antibodies recognizing Rb. (D) H1299 cells were transfected with the indicated plasmid DNAs for 48 h, and whole-cell extracts were immunoblotted as indicated using the appropriate antibodies.

E2F is negatively regulated by interactions with Rb and other “pocket” proteins that repress transcriptional activity (reviewed in reference 44). Hyperphosphoryation of Rb inhibits such interactions to activate E2F. In the case of wild-type adenovirus infection, E1A products bind pocket proteins to free E2F, which is thought to promote Rb hyperphosphorylation. To determine if the E4orf6/E1B55K complex affects the phosphorylation status of Rb, extracts from wild-type- and vector-infected cells were examined by Western blotting using anti-Rb antibodies that recognize all forms of Rb. Figure 7A shows that infection by AdE4orf6 alone had no effect on the gel migration of Rb; however, a distinct slowing in Rb migration consistent with hyperphosphorylation occurred in wild-type- and AdE4orf6/AdE1B55K-infected cells, although larger amounts of the slowest-migrating form were present with wild-type virus. With AdE1B55K alone, some effect on Rb migration was noted, but again as discussed above, such cells contain low levels of E4orf6 expressed from the vector backbone. Figure 7B shows that, following transfection with plasmid DNAs expressing E4orf6 and E1B55K, a similar shift in gel migration was observed, an effect not seen using the E4orf6-dBC mutant that does not form the ligase complex. Figure 7C shows that treatment of cell extracts with lambda phosphatase resulted in elimination of all slower-migrating bands to yield a single faster-migrating species, indicating that the migration shifts in Rb evident in Fig. 7 could be attributed entirely to changes in levels of phosphorylation. The effects of E4orf6 and E1B55K on Rb phosphorylation were also evident in IMR-90 cells, as seen above in Fig. 3B. Studies were also conducted to determine if similar effects could also be detected with the other pocket proteins. Figure 7D shows that coexpression of E4orf6 and E1B55K also induced slower-migrating forms of p130.

### E4orf6 protein binds to E2F transcription factors.

**FIG 8:**
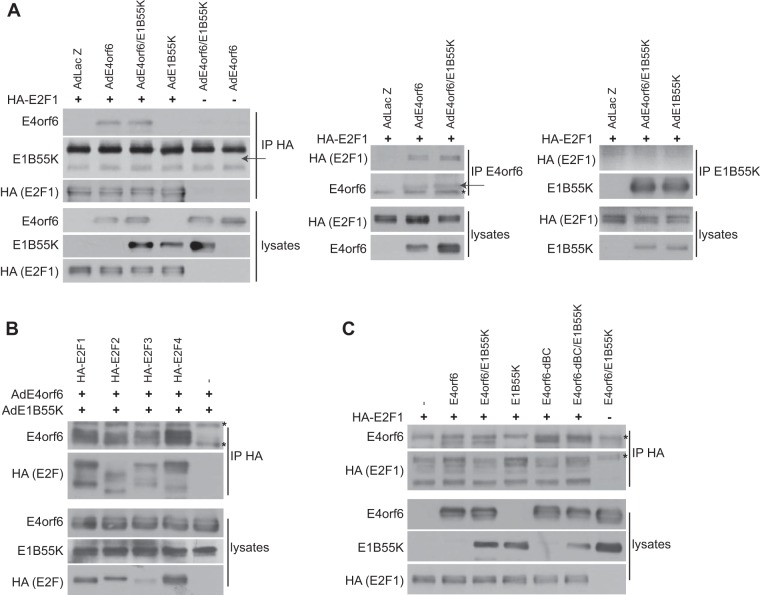
E4orf6 binds E2F1-4. (A) H1299 cells were infected with the viral vectors indicated at an MOI of 35 and complemented with AdLacZ as required for a total MOI of 70 and transfected with plasmid DNA expressing HA-E2F1 as indicated for 48 h. Immunoprecipitates obtained using antibodies against HA (E2F1 [left panel]), E4orf6 (middle panel), or E1B55K (right panel) as well as whole-cell extracts were immunoblotted as indicated using the appropriate antibodies. Arrows indicate the positions of migration of E4orf6 and E1B55K (if present). (B) H1299 cells were infected with AdE4orf6 and AdE1B55K at an MOI of 35 each and transfected with plasmid DNAs expressing the indicated E2F species for 48 h. Immunoprecipitates obtained using anti-HA (E2F) antibodies as well as whole-cell extracts were immunoblotted as indicated using the appropriate antibodies. (C) H1299 cells were transfected with the indicated plasmid DNAs for 48 h. Immunoprecipitates obtained using anti-HA (E2F1) antibodies as well as whole-cell extracts were immunoblotted as indicated using the appropriate antibodies. An asterisk denotes a background band.

To examine the mechanism of E2F activation, studies were conducted to determine if E4orf6 or E1B55K interacts with E2F. Cells were transfected with plasmid DNAs expressing human hemagglutinin (HA)-E2F1 and infected with Ad vectors expressing E4orf6 and E1B55K, alone or in combination, extracts were immunoprecipitated using anti-HA antibodies, and precipitates were examined by Western blotting using anti-HA, anti-E4orf6, or anti-E1B55K antibodies. Figure 8A (left panel) shows that while no E1B55K was evident in any of the immunoprecipitates (the arrow indicates the expected position), E4orf6 was present in that from cells expressing both E4orf6 and E1B55K, as well as with E4orf6 alone, suggesting that the E4orf6 protein interacts with E2F1. Using a different approach, immunoprecipitates were prepared from these cultures using either anti-E4orf6 or anti-E1B55K antibodies and then analyzed by Western blotting using anti-HA antibodies. Figure 8A (right panel) shows little coimmunoprecipitation of HA-E2F1 with E1B55K in either the presence or absence of E4orf6; however, in the case of E4orf6 (Fig. 8A, middle panel), HA-E2F1 clearly coimmunoprecipitated with E4orf6. These results confirmed that E4orf6 interacts with E2F1, although such interactions were not detected in a previous study (45). To determine if E4orf6 interacts with other members of the E2F family, a similar study was conducted in which cells infected by AdE4orf6 and AdE1B55K were transfected with plasmid DNAs expressing HA-tagged versions of human E2F1, E2F2, E2F3, and E2F4, and immunoprecipitates prepared using anti-HA antibodies were examined by Western blotting for coimmunoprecipitation of either E1B55K or E4orf6 protein. Figure 8B shows that all forms of E2F associated with E4orf6. Although results in Fig. 7B indicated that the E4orf6-dBC mutant protein was unable to induce the hyperphosphorylation of Rb, Fig. 8C shows that this species was nevertheless able to bind E2F1. These results suggested that hyperphosphorylation of Rb relied either on the presence of E1B55K or some other activity of the E4orf6-based ligase complex. It is important to note that thus far we have been unable to demonstrate convincingly the presence of E1B55K in complexes containing E2F and E4orf6 (Fig. 8A) (data not shown; see Discussion).

### The E4orf6/7 protein does not induce Rb phosphorylation and DNA synthesis.

**FIG 9:**
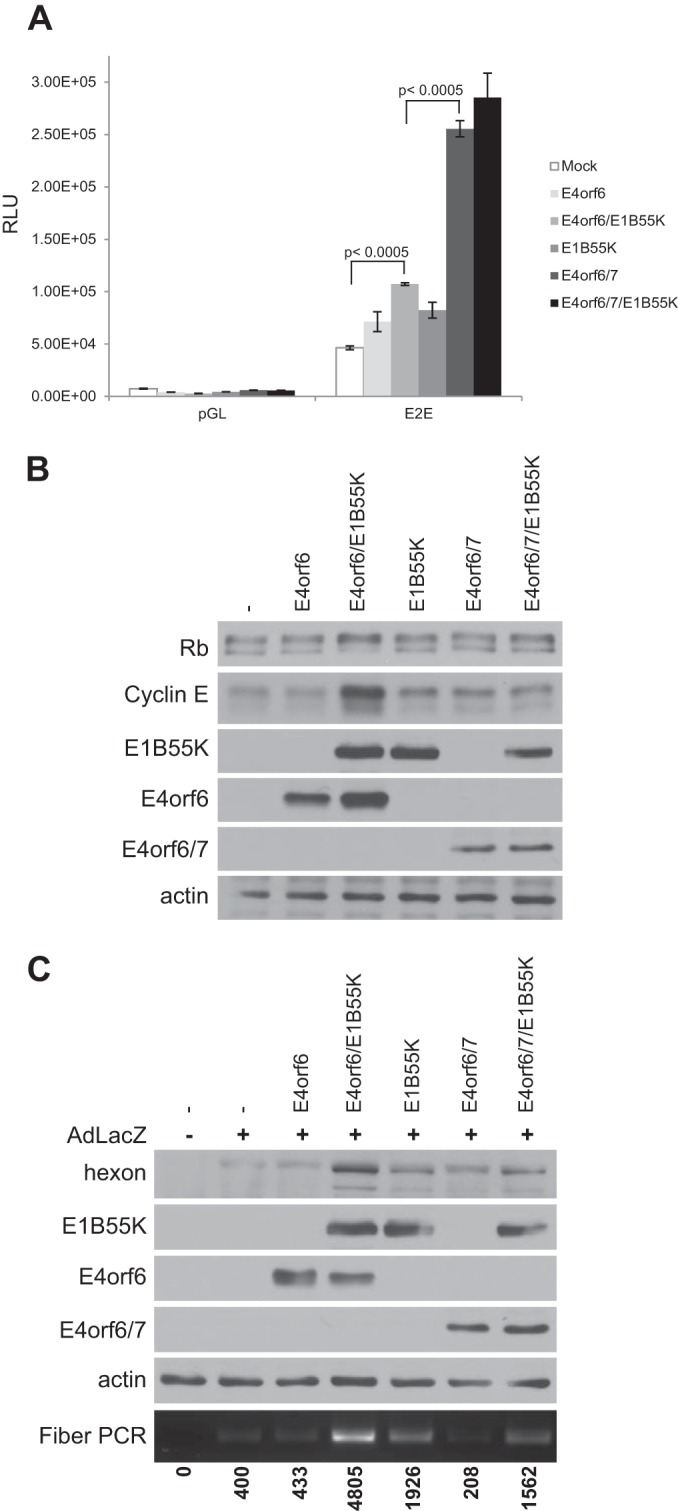
E4orf6/7 is not involved in the effects of E4orf6/E1B55K. (A) H1299 cells were transfected with control or E2E luciferase reporter construct, the *Renilla* luciferase control, and the indicated plasmid DNAs for 24 h. Lysates were used for luciferase assays. RLU, relative luminescence units. (B) H1299 cells were transfected with plasmid DNAs as indicated (the same amounts of DNA expressing E4orf6 and E4orf6/7 as in panel A) for 48 h. Whole-cell extracts were immunoblotted as indicated using the appropriate antibodies. (C) H1299 cells were infected with AdLacZ at an MOI of 70 and transfected with the indicated plasmid DNAs for 48 h. Whole-cell extracts were immunoblotted as indicated using the appropriate antibodies, and PCR for the fiber gene was performed. Numbers below the panel indicate quantification of the PCR bands by ImageJ.

Before discussing further the status of the E2F/Rb complex and the mechanism of activation of E2F activity and viral replication by E4orf6/E1B55K, we first considered the possibility that the adenovirus E4orf6/7 protein, which shares partial homology with E4orf6 and which is known to bind and potentiate E2F activity, might mimic the effects of E4orf6. We believed that the E4orf6 interaction with E2F involves a unique region of E4orf6 as the E2F-binding sequence utilized by E4orf6/7 is absent in E4orf6 (45). Nevertheless it was possible that E4orf6/7 might mimic at least some of the effects of E4orf6. To compare effects on E2F-dependent transcription, cells were transfected with plasmid DNA expressing a luciferase reporter driven by the E2E promoter and various combinations of cDNAs expressing E4orf6/7, E4orf6, or E1B55K in assays similar to those described in Fig. 5A. Figure 9A shows again that E4orf6 and E1B55K in combination, but not individually, induced E2E-dependent transcription; however, E4orf6/7 was even more effective, in either the presence or absence of E1B55K, consistent with a previous observation that a viral vector expressing E4orf6/7 from the CMV promoter induced expression of DBP (46). As E4orf6/7 does not bind E1B55K (data not shown), it was not surprising that expression of E1B55K did not further increase the promoter activity induced by E4orf6/7. To determine the effects on Rb phosphorylation and E2F-dependent transcription, cells were transfected with plasmid DNAs expressing various combinations of E4orf6/7, E4orf6, or E1B55K, and extracts were assessed by Western blotting for Rb gel migration and expression of the E2F-dependent cyclin E. Figure 9B shows that E4orf6/E1B55K induced a change in Rb gel migration typical of hyperphosphorylation and an increase in cyclin E expression, while E4orf6/7/E1B55K resulted only in a very slight increase of the hyperphosphorylated Rb species with no corresponding decrease in the hypophosphorylated Rb form and no increase in cyclin E expression. Finally, to determine the ability of E4orf6/7 to induce viral replication, studies similar to those in Fig. 2B were performed using the semiquantitative PCR-based assay for viral fiber DNA and Western blotting for late viral proteins. Figure 9C shows that expression of E4orf6 and E1B55K yielded results similar to those shown in Fig. 2B and 4B, but E4orf6/7 in the presence or absence of E1B55K had little effect. Thus, E4orf6/7 did not appear to play a role in the induction of viral replication seen in earlier studies (see Discussion).

### E4orf6 with E1B55K induces the release of E2F from the E2F-Rb complex.

**FIG 10:**
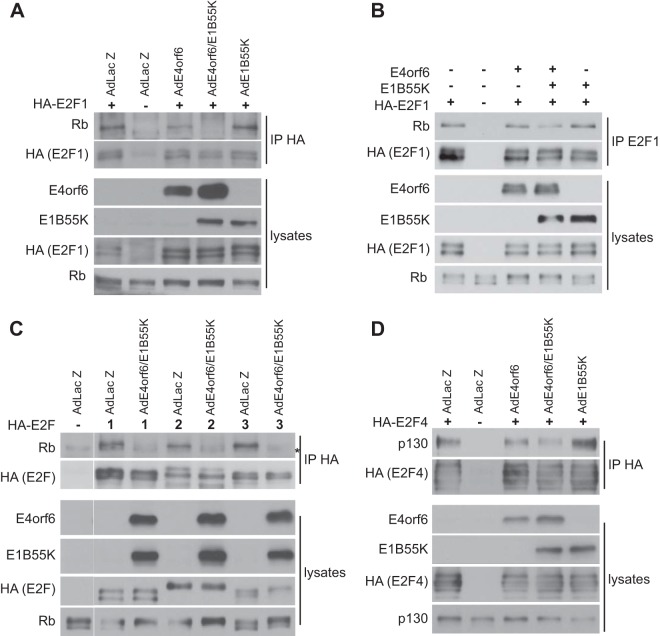
E4orf6/E1B55K disrupts interaction of pocket proteins with E2F. (A) H1299 cells were infected with AdE4orf6 and AdE1B55K at an MOI of 35 each or AdLacZ at an MOI of 70 and transfected with plasmid DNA expressing E2F1 as indicated for 24 h. Immunoprecipitates obtained using anti-HA (E2F1) antibodies as well as whole-cell extracts were immunoblotted as indicated using the appropriate antibodies. (B) H1299 cells were transfected with the indicated plasmid DNAs for 24 h. Immunoprecipitates obtained using anti-E2F1 antibodies as well as whole-cell extracts were immunoblotted as indicated using the appropriate antibodies. (C) H1299 cells were infected with AdE4orf6 and AdE1B55K at an MOI of 35 each or with LacZ at an MOI of 70 and transfected with the plasmid DNAs expressing E2F1-3 as indicated for 48 h. Immunoprecipitates obtained using anti-HA (E2F) antibodies as well as whole-cell extracts were immunoblotted as indicated using the appropriate antibodies. (D) H1299 cells were infected with AdE4orf6 and AdE1B55K at an MOI of 35 each or AdLacZ at an MOI of 70 and transfected with plasmid DNA expressing E2F4 as indicated for 48 h. Immunoprecipitates obtained using anti-HA (E2F4) antibodies as well as whole-cell extracts were immunoblotted as indicated using the appropriate antibodies. An asterisk denotes a background band.

To determine if the E4orf6/E1B55K complex induces the separation of the E2F/Rb complex in concert with Rb hyperphosphorylation, extracts from H1299 cells transfected with plasmid DNA expressing HA-E2F1 and infected with AdE4orf6 and/or AdE1B55K were treated with anti-HA antibodies, and the immunoprecipitates were analyzed by Western blotting using an anti-Rb antibody. Figure 10A shows that only in the case of coexpression of E4orf6 and E1B55K did Rb not coimmunoprecipitate with HA-E2F1. Figure 10B shows a study in which E4orf6 and E1B55K were expressed from transfected plasmid cDNAs. In this case, a clear reduction in the Rb-E2F1 interaction was also seen. Figure 10C shows that E4orf6/E1B55K also disrupted the Rb complex formed with E2F2 and E2F3. Figure 10D shows that the interaction of p130 with E2F4 was reduced by E4orf6/E1B55K, suggesting that some of the complexes were disrupted. Thus, the E4orf6/E1B55K complex appeared to stimulate E2F activity by inducing both hyperphosphorylation of Rb and p130 and separation of the E2F/Rb and E2F/p130 complexes, leading to E2F-dependent transcription, induction of viral and cellular DNA replication, and formation of early and late viral products accompanied by production of viral progeny. In total, the data from this extensive series of studies indicated that the E4orf6/E1B55K ligase complex appears to share several previously unidentified functional properties that mimic those of E1A products.

## DISCUSSION

In the present report, we have described how coinfection of viral vectors expressing E4orf6 and E1B55K resulted in replication of the E1A/E1B-deleted viruses, an effect that correlated with the activation of the E2E viral promoter through an E2F-dependent process. We found that in many ways the E4orf6/E1B55K complex mimics the effects of E1A products in that it induces the hyperphosphorylation of Rb family members and induces E2F-dependent transcription by uncoupling E2F transcription factors from Rb.

We have noted that the effects of E4orf6/E1B55K on protein expression (i.e., synthesis of DBP, late proteins, etc.) appear stronger than the effects seen by our luciferase reporter assay; however, we believe that measurements of protein levels (direct observations) are more representative of the true impact of the complex than are those detected by luciferase reporter assays (indirect observations).

We also believe that the E4orf6/7 protein, which shares partial homology with E4orf6, does not contribute significantly to the effects observed on E2F. In our studies, E4orf6/7 was sufficient to induce expression from the E2E promoter, but not the other effects observed with E4orf6/E1B55K. These findings were consistent with results from Hemstrom et al. (47) in which a viral mutant expressing E4orf6/7 as the only E4 product was found to activate E2 genes normally but could not sustain viral DNA replication, whereas a mutant expressing both E4orf6/7 and E4orf6 induced viral DNA replication normally, suggesting that E4orf6 or the E4orf6/E1B55K complex plays another role in DNA replication. These findings, however, do not appear consistent with results of studies by O’Connor and Hearing (46) in which they observed that an E1A-deleted virus overexpressing E4orf6/7 from a CMV promoter was even better at viral replication than the effects we report here with E4orf6/E1B55K. It should be noted that the virus used in this previous study was not deleted in the E1B region and contained E4orf6 coding sequences in its backbone. As there is a low level of expression from the E4 region in the absence in E1A, it can be expected that some expression of the E4orf6/E1B55K ligase in addition to the high level of expression of E4orf6/7 probably took place, possibly explaining the higher level of virus replication they observed. E4orf6/7 was shown to relocalize E2F4 to the nucleus and induce E2 transcription, a function that requires both the protein region unique to E4orf6/7 that binds E2F and the nuclear localization signal (NLS) motif present in the sequence shared with E4orf6 (48). Nevertheless we found that these effects of E4orf6/7 were not sufficient to induce viral DNA synthesis. E4orf6/7 activates only a specific class of E2F-dependent promoters—those containing two inverted E2F binding sites, as are present in the E2 and E2F1 promoters (49, 50). Thus, E4orf6/E1B55K may activate a broader range of E2F-responsive promoters, which may be essential for DNA synthesis. Alternatively, E4orf6/E1B55K may contribute additional as yet unknown functions to promote DNA synthesis—perhaps the ability we observed of E1B55K acting alone but not E2F1 to activate the E2L promoter.

Most importantly, we have described a new activity of the Ad5 E4orf6/E1B55K ubiquitin ligase—that of promoting viral infection by activating E2F. Although it has been known for some time that E1A proteins are the chief activators of E2F to allow efficient viral replication, our current findings indicated that when expressed at high levels, the E4orf6/E1B55K complex possesses a similar ability, albeit at considerably lower efficiency. At present, we understand little about the mechanism of this function. Previous studies with E4orf6/E1B55K mutants focused largely on effects related to the degradation of components of the DNA repair pathway to prevent formation of viral DNA concatamers and the regulation of late viral mRNA transport, and few addressed effects on E2F. Our findings are consistent with those of a previous study showing that E1B55K is required for induction of the E2F-regulated expression of cyclin E and hyperphosphorylation of Rb (51–53); however, this work did not consider either the more global role of the E4orf6/E1B55K ligase complex or the mechanism of E1B55K involvement. As the effects we observed require formation of the ligase complex, it seems possible that degradation of one or more critical regulatory targets might be involved. Degradation of p27, an inhibitor of Cdks, seemed a possible candidate; however, p27 did not appear to be affected (data not shown). The interaction of E4orf6 protein with E2F suggested that such binding might target the ligase to E2F-Rb complexes, thus presenting potentially key substrates for degradation, including components of complexes associated with Rb that repress E2F-dependent transcription (54–68). We have examined three such species—histone deacetylase 1 (HDAC1), HDAC2, and retinoblastoma-binding protein 1 (RBP1); however, none was found to be significantly degraded (data not shown). It is also possible that these effects involve one of the other known functions of E1B55K, including transcriptional repression as seen in the case with p53 (69–71), its role as a SUMO ligase (72, 73), or its ability to bind promyelocytic leukemia (PML) proteins (74). In these cases, E4orf6 would function to target E1B55K to E2F/Rb complexes, although as pointed out earlier, we have not been successful thus far in demonstrating the presence of E1B55K in E2F/E4orf6 complexes. It has been shown that the E4orf6 ligase complex of Ad12, but not other adenovirus serotypes, including Ad5, degrades the E2F-associated protein TOPBP1 (16, 17), and thus degradation of an as yet unidentified substrate of this type might play some role. In this case, E1B55K might contribute through one of its other functions unrelated to the ligase *per se*.

The present studies clearly indicate that when expressed at high levels in H1299 cells, E4orf6 and E1B55K can mimic many of the effects of E1A on E2F; however, when these proteins are expressed at lower levels in the context of a normal viral infection, does this activity contribute in any way to the replication of wild-type virus expressing E1A? These issues are addressed in the accompanying article (75).

## MATERIALS AND METHODS

### Cell lines, plasmids, and viruses.

Human non-small-cell lung carcinoma H1299 cells (ATCC CRL-5803), embryonic kidney 293 cells (HEK-293; ATCC CRL-1573), and IMR-90 cells (ATCC CCL-186) were grown in Dulbecco’s modified Eagle’s medium (Gibco) without antibiotics and supplemented with 10% fetal bovine serum (Multicell) at 37°C in 5% CO_2_. IMR-90 cells were left for 48 h at confluence before infection.

The plasmids used in the studies were pcDNA3-E4orf6 (76), the pcDNA3-E4orf6 L47G L122S double BC mutant (6), pcDNA3 E4orf6/7 (77), pcDNA3-E1B55K (78), and pcDNA3-HA-E2F1 (kindly provided by Doron Ginsberg)**.** The HAdV5 promoter constructs are based on the pGL3-basic vector (Invitrogen) as described previously (79). The pGL-E2F reporter and pGL3 vectors were kindly provided by Joe Mymryk (41). pCMV-HA-E2F1, -HA-E2F2, -HA-E2F3, and -HA-E2F4 were all provided by Fred Dick.

Adenoviral vectors and wild-type (WT) virus used have been described previously: AdE1B55K (22, 80), AdE4orf6 (22, 80), AdLacZ (81), and H5*pg*4100 (WT) (82).

### Antibodies and reagents.

The rabbit polyclonal antibodies used were E4orf6 (1807) (83), p130 C-20 (catalog no. Sc-317; Santa Cruz), Ad5 capsid (84), and E4orf4 polyclonal 2419 (85). Antibodies against adenovirus polymerase were kindly provided by Ron Hay. The following mouse monoclonal antibodies were used: Ad5 E1B55K 2A6 (86), E2A DNA-binding protein B6-8 (87), actin C4 (catalog no. 691001; MP Biomedicals); Ad5 E4orf6/E4orf6/7 RSA3 (88), IgG2a (M5409; Sigma-Aldrich), Ad5 hexon 2Hx-2 (HB-8117; ATCC), Rb OP66 (catalog no. OP66; Millipore) or 4H1 (catalog no. 9309; Cell Signaling), cyclin A BF683 (catalog no. Sc-239; Santa Cruz), Cdc6 0.T.17 (catalog no. Sc-70826; Santa Cruz), HA tag HA.11 clone 16B12 (catalog no. MMS-101R; Covance), E2F1 KH95 (catalog no. Sc-251; Santa Cruz), Ad5 E1A M58 (from Roger Grand) and M73 (89), and cyclin E HE12 (catalog no. sc-241; Santa Cruz). The rat monoclonal antibodies were E1B55K 4E8 (90) and E4orf3 6A11 (91). Horseradish peroxidase (HRP)-conjugated secondary antibodies for detection in Western blotting were goat anti-mouse IgG, goat anti-rabbit IgG, and goat anti-rat IgG (Jackson ImmunoResearch Laboratories) and rat anti-mouse κ light-chain-specific clone 187.1 (catalog no. 559751; BD Biosciences). Fluorophore-conjugated secondary, anti-mouse Alexa Fluor 488 (A-11029) was from Invitrogen. Additional reagents included DAPI (4′,6-diamidino-2-phenylindole; Invitrogen), propidium iodide (catalog no. PPI777; BioShop), paraformaldehyde (Electron Microscopy Sciences), Lipofectamine 2000 reagent (Invitrogen), the Promega dual luciferase reporter assay system (catalog no. E1910), lambda protein phosphatase (catalog no. P0753; New England Biolabs), and polyethyleneimine (PEI) (catalog no. 23966; Polysciences, Inc.).

### DNA transfections and infections.

DNA transfections (except for dual luciferase reporter assays) were done using Lipofectamine 2000 reagent according to the manufacturer’s recommendations. The final amounts of DNA per well were equalized by addition of vector plasmid pcDNA3. For DNA transfections in the dual luciferase reporter assays, medium was first replaced with fresh Dulbecco’s modified Eagle’s medium (DMEM) plus 10% fetal bovine serum (FBS) 1 h before transfection. DNA was incubated with 25-kDa linear polyethyleneimine (PEI) at a ratio of 1 µg of DNA to 12 µl of PEI to 600 µl of DMEM for 30 min at room temperature as described previously (15). Medium was removed from cells, and the DNA-PEI mixture was added to the cells for 5 to 6 h before being replaced by fresh medium. For infections, cells were infected with viruses diluted in infection medium (0.2 mM CaCl_2_, 0.2 mM MgCl_2_, and 2% serum in phosphate-buffered saline [PBS]) for 90 min before removal and the addition of normal growth medium. A multiplicity of infection (MOI) of 35 PFU per cell was used for viral vectors, and the final amounts of vectors were adjusted to be equal by the addition of AdLacZ. Infections with WT virus were done at 1 FFU/cell for immunofluorescence or 5 FFU/cell for control infections. For infections and cotransfections, cells were first infected at the indicated MOI for 90 min and then transfected with indicated plasmid DNAs.

### Protein extraction.

Cells were washed with PBS and removed at different times postinfection/posttransfection by incubation for 5 min with gentle agitation with 0.53 mM EDTA and collected by centrifugation. For evaluation of viral gene expression (Fig. 1), cell pellets were incubated for 5 min on ice in CHAPS {3-[(3-cholamidopropyl)-dimethylammonio]-1-propanesulfonate} buffer (4% CHAPS [BioShop], 30 mM Tris-HCl [pH 8.5], 50 mM NaCl) plus inhibitors (protease inhibitor cocktail [Sigma], 1 mM Na_3_VO_4_, 10 mM NaPPi, 10 mM NaF). Cells were then lysed by sonication, and protein concentrations of the lysates were determined using Bio-Rad DC protein assay reagents according to the manufacturer’s protocol. Extracts were brought to 7 M urea and 2 M thiourea (GE Healthcare Biosciences) and clarified by centrifugation at 13,000 × *g* for 5 min, and protein concentrations were recalculated according to volume. For viral DNA amplification of extracts and other protein blotting, cells were lysed for 20 min on ice in the lysis buffer (50 mM Tris-HCl [pH 8.0], 150 mM NaCl, 5 mM EDTA, 1% NP-40, 0.1% SDS, 0.1% Triton X-100, protease inhibitor cocktail [Sigma], 1 mM Na_3_VO_4_, 10 mM NaPPi, 10 mM NaF) and clarified by centrifugation at 13,000 × *g* for 10 min. For immunoprecipitation studies, cells were lysed for 20 min on ice in lysis buffer (20 mM Tris-HCl [pH 7.5], 150 mM NaCl, 2 mM EDTA, 1% Triton X-100, 5% glycerol, protease inhibitor cocktail [Sigma], 1 mM Na_3_VO_4_, 10 mM NaPPi, 10 mM NaF) and clarified by centrifugation at 13,000 × *g* for 10 min. For the lambda phosphatase treatment, cells were lysed for 20 min in phosphatase buffer (30 mM Tris-HCl [pH 7.5], 150 mM NaCl, 0.5% NP-40, protease inhibitor cocktail [Sigma]) and clarified by centrifugation at 13,000 × *g* for 10 min. The protein concentrations of the lysates were determined using Bio-Rad DC protein assay reagents according to the manufacturer’s protocol. Extracts were adjusted to 1× MnCl_2_ (10× solution provided with the enzyme) and 4,000 U/ml lambda phosphatase protein and incubated at 30°C for 30 min. The reaction was stopped by the addition of an equal volume of 2× SDS-PAGE sample buffer, and protein concentrations were recalculated according to volume.

### Immunoblotting.

Equal amounts of proteins were separated by SDS-PAGE and then transferred to polyvinylidene difluoride membranes (Millipore) blocked using 5% skimmed milk prior to antibody blotting. Primary antibodies were added to the membranes for 2 to 3 h at room temperature or overnight at 4°C. Membranes were washed with PBS containing 0.1% Tween 20, and the secondary antibody was added for 1 h at room temperature. Detection was performed using Western Lightning Chemiluminescence Reagent Plus (PerkinElmer).

### Viral DNA amplification.

H1299 cells were infected with either wild-type virus, Ad vectors expressing the appropriate combination of E4orf6 and E1B55K, or AdLacZ vector at an MOI of 70 cotransfected with the indicated plasmids for 24 to 48 h at 37°C as indicated. Cell extracts were made as described above and treated with proteinase K for 1 h at 55°C. Viral DNA content was measured by PCR with primers specific for the Ad5 fiber gene (forward, CACCCCTCACAGTTACCTCAGAAGCCC and reverse, GTCTGTTTTGAGAATCAATCCTTAGTCCTC). PCR products were visualized on 1.2% agarose gels. Purified adenovirus DNA was used as a positive control for PCR analyses.

### Measurement of progeny virions.

H1299 cells were infected with AdLacZ vector at an MOI of 35 and cotransfected with the indicated plasmids for 48 h 37°C. Cells were pelleted, lysed by 4 cycles of freezing and thawing, and clarified by centrifugation at 1,900 × *g* for 10 min. Virus titers in the supernatant were determined by plaque assay in HEK293 cells. A representative experiment is shown, with error bars from an average of three independent plaque assays.

### Immunoprecipitations.

To examine interactions between E2F proteins, E4orf6 and E1B55K, coimmunoprecipitation studies were done in which cells were infected with the appropriate combinations of adenovirus vectors and transfected with plasmid DNAs expressing HA-E2F1, HA-E2F2, HA-E2F3, or HA-E2F4 for 48 h, and then protein extracts were made as described above. Between 200 and 500 µg of proteins was precleared overnight at 4°C with protein G Sepharose beads (GenScript). Beads were removed by centrifugation, and supernatants were incubated with either anti-E4orf6 polyclonal antibody 1807, anti-E1B55K rat monoclonal antibody 4E8, or anti-HA mouse monoclonal antibody, followed by incubation with protein G Sepharose (GenScript). The beads were extensively washed in lysis buffer and examined by SDS-PAGE. For interactions between E2F proteins and Rb or p130, cells were infected with an appropriate combination of vectors and transfected with plasmid DNAs expressing HA-E2F1 or HA-E2F4 for 24 h. Alternatively, cells were transfected with the appropriate combination of pcDNA3-E4orf6, pcDNA3-E1B55K, and pcDNA3 HA-E2F1 for 24 h. Immunoprecipitations were performed as described above using the anti-HA or anti-E2F1 KH95 mouse monoclonal antibody. The detection of Rb and HA-tagged proteins by Western blotting with primary mouse monoclonal antibodies was done with secondary horseradish peroxidase (HRP)-conjugated rat anti-mouse κ light-chain-specific antibody.

### Luciferase reporter assay.

Dual luciferase reporter assays were performed according to the manufacturer’s recommendations (Promega) as reported previously (79). Briefly, H1299 cells were transfected as described above using 1 µg of reporter, effector plasmid DNAs, and 0.5 µg of pRL-TK (Promega), which expresses *Renilla* luciferase under the control of the herpes simplex virus thymidine kinase (HSV-TK) promoter. Total cell extracts were prepared 24 h after transfection, and firefly luciferase activity was assayed using 10 µl of lysed extract in a Lumat LB9507 luminometer (Berthold Technologies). All samples were normalized for transfection efficiency by measuring the *Renilla* luciferase activity.

### Immunofluorescence microscopy.

Cells grown on coverslips and infected as described above were washed once in PBS and fixed with methanol for 15 min at −20°C. Cells were incubated with primary E2A DNA-binding protein B6-8 antibody diluted in PBS for 2 h at room temperature and then washed two times for 5 min with PBS followed by incubation with secondary antibody and DAPI diluted in PBS for 1 h at room temperature in the dark. Coverslips were washed twice with PBS and once with water before being mounted on slides in mounting medium (Immu-Mount; Thermo Scientific). Images were acquired at the McGill Life Sciences Complex Imaging Facility on an LSM4 confocal microscope with a 63× objective using the ZEN 2011 Image browser software. Images were cropped using Adobe Photoshop CS3, the brightness was adjusted, and then the image was assembled with Adobe Illustrator CS3.

### Flow cytometry.

Cells were detached with trypsin, fixed in formalin, and permeabilized with 0.1% Triton X-100. One million cells were incubated with primary antibody (anti-hexon 2Hx-2) or isotype-matched normal mouse IgG2a) diluted in PBS–1% normal goat serum (NGS) for 1 h at room temperature. Cells were washed once and then incubated with anti-mouse antibody labeled with Alexa Fluor 488 for 30 min at room temperature in the dark. Cells were washed again, resuspended in PBS, and analyzed immediately by flow cytometry on a FACSCalibur flow cytometer using CellQuest software (Becton, Dickinson, Oxford, United Kingdom). Data were analyzed using FCS Express software (De NoVo Software). For cell cycle analysis, cells were grown in six-well plates and infected as described above with the appropriate combination of vectors for 48 h. Cells were lifted with trypsin and washed twice with PBS, and 1 million cells were fixed/permeabilized in 80% ethanol for 1 h at −20°C. Cells were spun down and treated with RNase A before being transferred in 96-well plate in which propidium iodide was added at a final concentration of 5 µg/ml. Cells were analyzed by flow cytometry on a Guava Easycyte HT flow cytometer (Millipore), and data were processed using FlowJo software (Tree Star, Inc.).
